# Effect of gonococcal lipooligosaccharide variation on human monocytic cytokine profile

**DOI:** 10.1186/1471-2180-7-7

**Published:** 2007-01-26

**Authors:** Julia B Patrone, Daniel C Stein

**Affiliations:** 1 Department of Cell Biology and Molecular Genetics, University of Maryland, College Park, MD 20742 USA

## Abstract

**Background:**

*Neisseria gonorrhoeae *is an obligate human pathogen that causes significant worldwide morbidity. *N. gonorrhoeae *expresses lipooligosaccharide (LOS), a phase variable molecule that plays an important role during pathogenesis of the organism. Alteration in the structure of gonococcal LOS correlates with altered disease presentation. In addition, LOS sialylation occurs readily *in vivo*, though the role of this sialylation during disease is unknown.

**Results:**

Challenge of human monocytes with purified LOS preparations isolated from strains expressing distinct structurally defined LOSs resulted in identical production of the proinflammatory cytokines tumor necrosis factor alpha (TNFα) and interleukin-12 (IL-12). Similar results were seen when monocytes were challenged with either live or gentamicin-killed whole cell gonococcal variants expressing these LOS structures, although greater cytokine production was observed in comparison with challenge by purified LOS. Challenge of a human primary monocyte model with distinct LOS variants resulted in similar production of TNFα, IL-12, interleukin-10 (IL-10), and interleukin-8 (IL-8). A cytokine array was employed to allow measurement of a broad range of cytokines in samples challenge with gonococcal LOS variants as well as variants expressing sialylated LOS. Challenge of primary monocytes with sialylated gonococci was shown to elicit the production of more MCP-2 (monocyte chemoattractant protein-2) in comparison with challenge by unsialylated gonococci.

**Conclusion:**

We demonstrated that while alterations in the carbohydrate moiety of LOS do not impact the production of most cytokines by human monocytes, whole-cell bacterial challenge is more stimulatory than challenge with purified LOS, implying that other gonococcal cell surface antigens are important for the elicitation of cytokines. Challenge with gonococci expressing sialylated LOS resulted in elicitation of more of the chemokine MCP-2 from challenged cells in comparison with gonococci expressing unsialylated LOS. As MCP-2 is an important chemoattractant, this indicates that *in vivo *sialylation may play an important role during the pathogenesis of *N. gonorrhoeae*.

## Background

*Neisseria gonorrhoeae *is an obligate human pathogen that has an important global impact on human health. Significant disease sequelae can result from infection by *N. gonorrhoeae*, including pelvic inflammatory disease (PID), which often results in sterility in women. This organism employs several cell surface virulence factors, including lipooligosaccharide (LOS), opacity-associated protein (Opa), and pili, to initiate infection. *N. gonorrhoeae *achieves colonization and invasion through a multi-step process, presumably using multiple surface molecules, and interacting with many types of host cells and host cell receptors (For recent review, see [1]). Inflammation and immune cell influx are two important diagnostic symptoms of gonorrhea.

**Figure 1:**
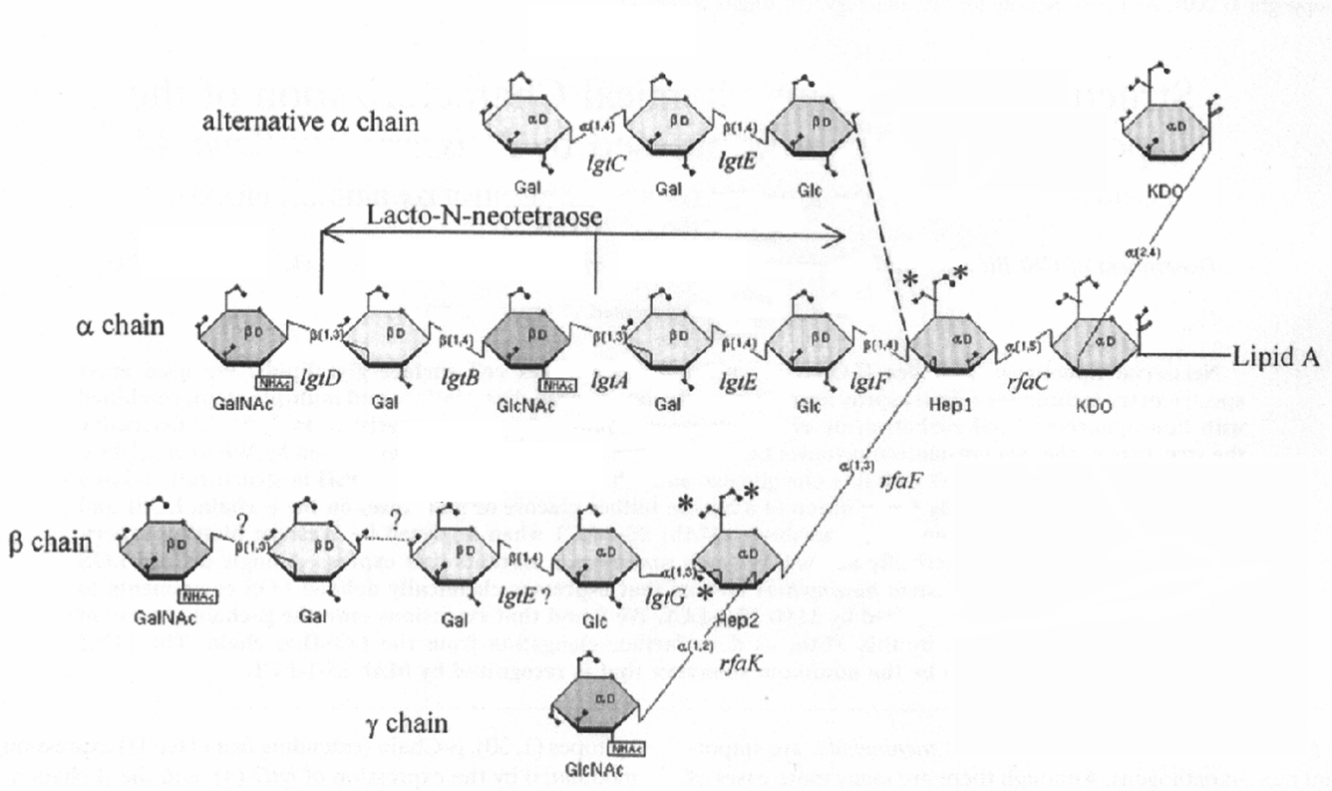
**Schematic representation of the structure of LOS from *N. gonorrhoeae *strain F62**. LOS biosynthesis genes are indicated by italics. Dotted lines indicate alternative LOS structures. Asterisks (*) indicate possible site of PEA or phosphate attachment. The broken line indicates the addition of an alternate α chain, joined to HepI as a β1-4 linkage (Figure modified from **[48]**).

Lipopolysaccharides (LPS) constitute a family of toxic glycolipids which are integral in the outer membranes of gram-negative organisms. These molecules are critical for the integrity and functioning of the outer membrane [2]. They are also important surface antigens and are highly immunostimulatory. Systemic distribution of LPS can lead to endotoxic shock [3]. In addition, variation in LPS structure has been shown to affect bacterial virulence [4]. An LPS molecule generally consists of a highly hydrophobic Lipid A, a covalently attached core region (which can be divided into an inner and outer core), and a polymer of a repeating saccharide. The O-polysaccharide portion of the molecule, however, is not ubiquitous. Several gram-negative bacterial strains, such as *N. gonorrhoeae*, express a glycolipid with a truncated, non-repeating oligosaccharide chain, called LOS (see Fig. 1 for schematic of gonococcal LOS) [5]. The gonococcus variably expresses some of the genes encoding the glycosyl transfersases needed for LOS biosynthesis, and this results in rapid and reversible alterations in the oligosaccharide structure [6]. A single strain of *N. gonorrhoeae *(and even a single gonococcal cell) can express several LOS structures at any given time [7].

The role of gonococcal LOS has been shown by many groups to be important in disease pathogenesis [8-10]. Harvey, *et al*. have demonstrated an important interaction between those gonococci expressing lacto-*N*-neotetraose-terminal LOS and the asialoglycoprotein (ASGP) receptor of primary human urethral epithelial cells [10]. Strains expressing lacto-*N*-neotetraose-terminal LOS were significantly more invasive than strains containing a deletion of the terminal Galβ1-4 and subsequent sugars [11]. Song, *et al*. demonstrated that in the absence of Opa proteins, strains expressing a specific LOS (containing a terminal lacto-*N*-neotetraose) promoted increased gonococcal invasion of epithelial monolayers [9]. In addition, gonococci expressing LOS with a predominantly specific carbohydrate structure have been associated with either symptomatology or a lack of symptoms in male clinical trials, further supporting the observation of pathogenic LOS phenotypes [8]. These studies demonstrated that after inoculating volunteers with low-molecular weight LOS variants, urethritis developed and primarily high molecular weight LOS (terminal lacto-*N*-neotetraose) was purified from the exudates. Thus, while the lipid A portion of the LOS molecule has historically been designated the immunogenic portion of the molecule, the carbohydrate structures of LOS also play an important function in disease pathogenesis.

Sialylation of LOS also impacts gonococcal interactions with host cells [12]. Sialylated strains have been shown to be resistant to complement-mediated killing [13], to have a decreased ability to induce oxidative burst in neutrophils[14], and to possess a decreased ability to adhere to neutrophils in the absence of complement [15]. It has also been observed that sialylation of gonococci *in vitro *[by the addition of cytidine 5'-monophospho-*N- *acetylneuraminic acid (CMP-NANA)] reduces their ability to invade tissue culture epithelial cells [16]. At present, further implications of LOS sialylation in the context of molecular mimicry remain unclear, though this process appears to be of significant biological importance. Since certain gonococcal LOS structures (potentially able to be sialylated) are associated with specific degrees of symptomatology, it is possible that LOS structure is integral in determining the severity of a gonococcal infection, and may serve to contribute to bacterial evasion of the host immune system.

Lipopolysaccharide (LPS) is known to be highly immunostimulatory, and can elicit a variety of pro-inflammatory cytokines and chemokines from host cells during infection [17]. However, the immunostimulatory effects of gonococcal LOS during infection remain somewhat controversial. Several studies differ in their observations regarding the types and amounts of cytokines elicited. Some of these contradictions may stem from use of different bacterial strains, phase variation of LOS molecules in culture, and different cellular models of infection, possibly resulting in different LOS receptor expression/density. In addition, the biological significance of LOS antigenic variation during infection has not been determined. In general, it is accepted that the fallopian tube tissue damage resulting from gonococcal infections can be attributed to the cytokine tumor necrosis factor alpha (TNFα) [18,19]. However, since epithelial cell-associated gonococci have evolved a mechanism to protect the cells from undergoing TNFα-mediated apoptosis [20], the role of TNFα in damage to other reproductive mucosa is unclear. In addition, interleukin-8 (IL-8), interleukin-6 (IL-6), and interleukin-1 beta (IL-1β) have been measured following gonococcal challenge of several models [21-25]. *In vivo *studies are rare, but occasionally naturally occurring infections in women have been monitored. Gonococcal cervicitis in female sex workers has been shown to cause increases in interleukin-4 (IL-4), IL-6, interleukin-10 (IL-10), and TNFα, as well as a decline in CD4^+ ^T cell counts [26]. Though the importance of the cytokine IL-12 during gonococcal infections remains to be determined, this cytokine appears to be of significance during meningococcal infections [27]. Cytokines such as TNF, IL-1β, IL-12, IL-10, MCP-1 (monocyte chemoattractant protein-1), IL-6, and RANTES (regulated upon activation, normal T-cell expressed, and presumably secreted) have also been measured during challenge with meningococcal LOS [27-30]. However, the ability of monomeric peptidoglycan fragments from *N. gonorrhoeae *to damage human fallopian-tube mucosa has also been demonstrated [31]

Different LOS structures have been shown to correlate with disease symptomatology: lacto-*N*-neotetraose LOS is associated with symptomatic gonococcal infection, while lactosyl LOS is associated with asymptomatic gonococcal infection [8]. In this study, we tested whether these naturally occurring LOS structures would differentially impact cytokine production by human monocytes. We hypothesized that compared with strains F62 and F62ΔlgtD (expressing lacto-*N*-neotetraose LOS), strain F62ΔlgtA (expressing lactosyl LOS) would elicit lower levels of the cytokines associated with symptoms (such as TNFα). We further hypothesized that this strain might elicit greater production of IL-10, thereby suppressing pro-inflammatory responses. The ability of purified LOS and isogenic strains expressing two distinct LOS variants to elicit cytokines was investigated. In addition, the effects of bacterial sialylation upon cytokine production were also examined. We chose to focus on measurement of the cytokines TNFα, IL-10, IL-12, and IL-8 due to both significance in published work and potential significance regarding the overall disease model. In our studies, both a human tissue culture cell model and a primary human cell model were utilized.

## Results

### Challenge of THP-1 cells with equivalent amounts of LOS/LPS from different variants results in similar production of TNFα and IL-12

**Figure 2:**
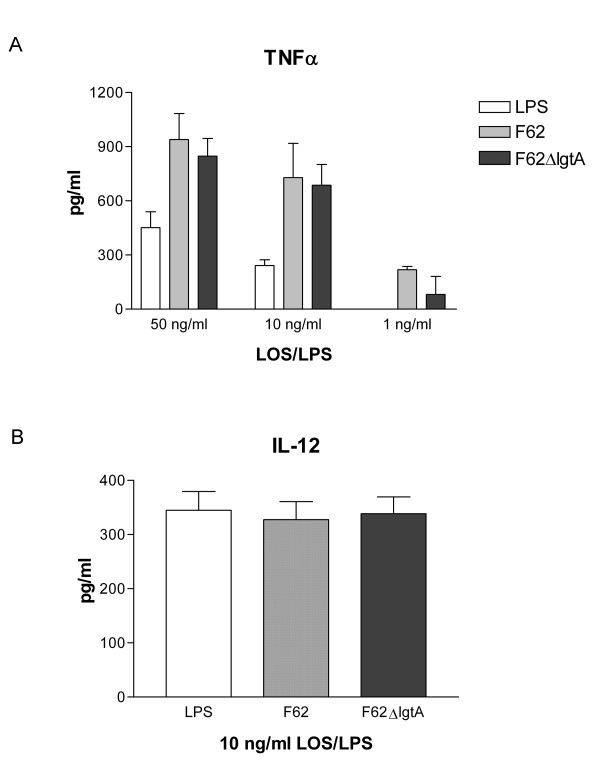
**Production of TNFα and IL-12 by THP-1 cells challenged with purified LOS/LPS**. (A) Sample supernatants were collected 7 hrs after challenge of THP-1 cells (1 × 10^6^/sample). These data are the result of two experiments, each performed in triplicate. Error bars indicate the standard deviation of the mean. (B) Sample supernatants were collected 16 hrs after challenge of THP-1 cells 1 × 10^6^/sample. These data represent one experiment, performed in triplicate, and the trend is similar to that seen from multiple single experiments, each performed at different time points. Error bars indicate the standard deviation of the mean. (LPS = *E. coli *LPS).

In order to directly study the effects of LOS variation on human monocytic cytokine production, THP-1 cells were challenged with purified preparations of LOS. Using *Escherichia coli *LPS as a positive control and unchallenged cells as a negative control, we were able to compare the immunostimulatory properties of LOS purified from wild type F62 with truncated LOS, purified from the F62ΔlgtA mutant. Analysis of cell supernatants by ELISA revealed no significant differences in the production of TNFα over a dose range, as seen in (Fig. 2A). For subsequent experiments, an initial challenge dose of 10 ng/ml LOS/LPS was used, as it did not result in significant monocyte cell death, yet did result in measurable cytokine production. Measurement of IL-12 similarly revealed no significant differences between F62 and the truncated mutant, as seen in (Fig. 2B). Unchallenged samples did not result in measurable cytokine production. IL-10 expression was also analyzed, but no values above the limit of detection by ELISA were obtained. While it does appear that gonococcal LOS elicits significantly more TNFα from THP-1 cells than does *E. coli *LPS (Fig. 2A) we attribute this to differences in the molar ratios of the two molecules, when used at the same concentration.

### Challenge with distinct whole-cell gonococcal LOS variants elicits similar levels of TNFα and IL-12

**Figure 3:**
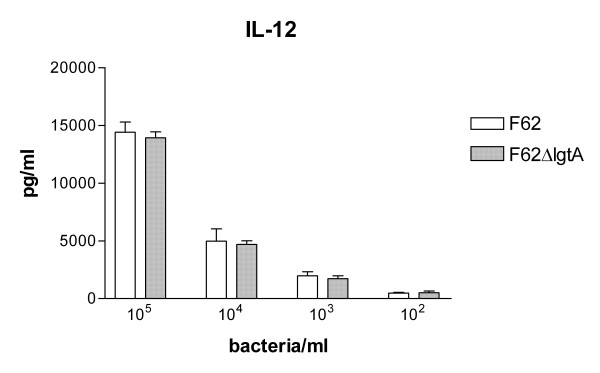
Production of IL-12 by THP-1 cells challenged with live gonococcal variants over a dose range. Various doses of live gonococci were added toTHP-1 cells (1 × 10^6^/ml). At 18 hours post infection, supernatants were collected and analyzed by ELISA. Data shown are from one experiment, performed in triplicate. Error bars indicate the standard deviation.

**Figure 4:**
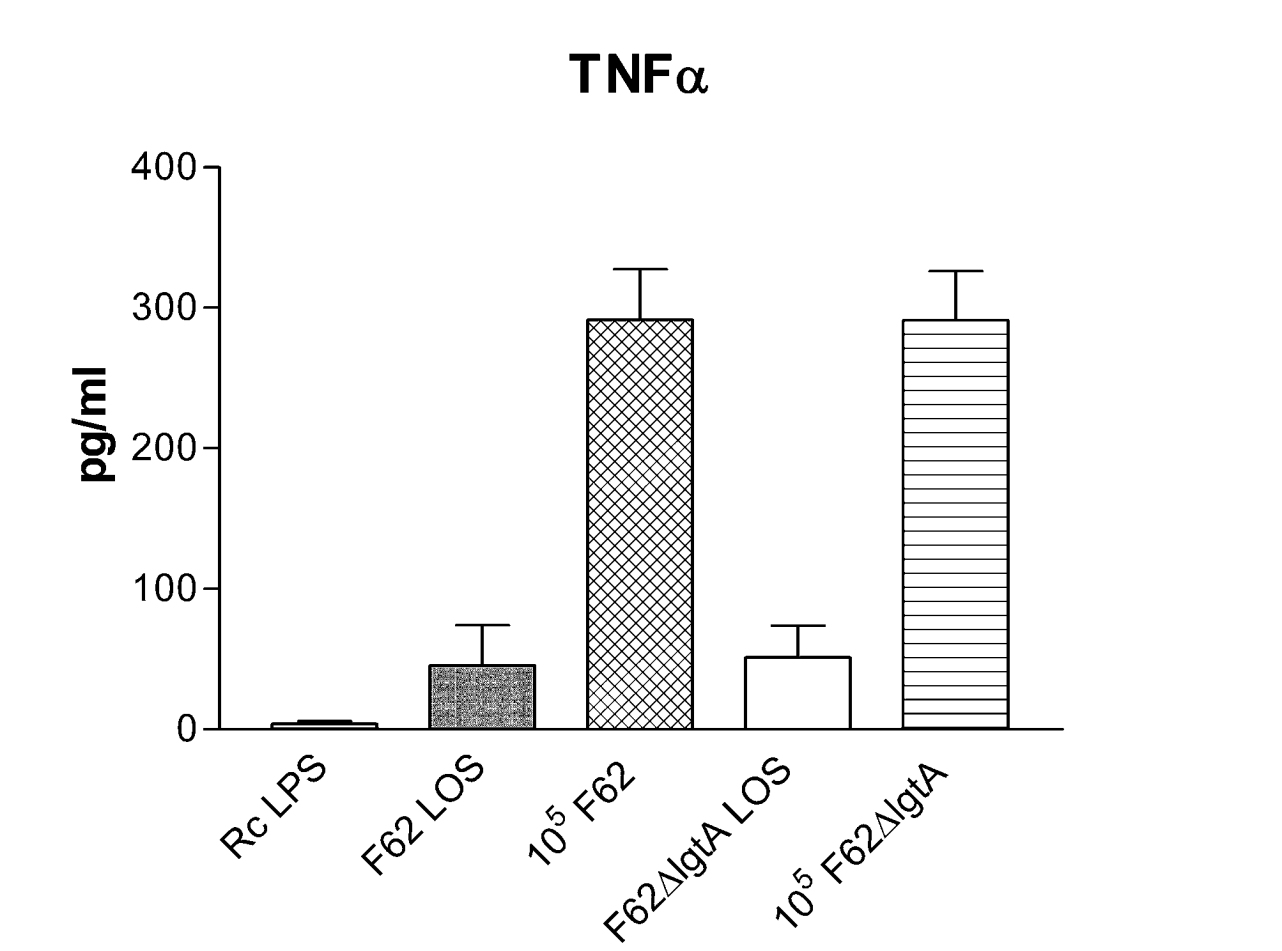
Comparison of TNFα production upon challenge with live gonococci vs. challenge with purified LOS. THP-1 cells (1 × 10^6^/ml) were challenged with either 1 × 10^5^/ml live gonococci or a molar equivalent amount of LOS/LPS (10 ng/ml). At three hours post challenge, sample supernatants were analyzed for TNFα by ELISA. These data represent two experiments, each performed in triplicate. Error bars indicate the standard deviation. (Rc LPS was purified from a rough LPS *E. coli *variant.)

**Figure 5:**
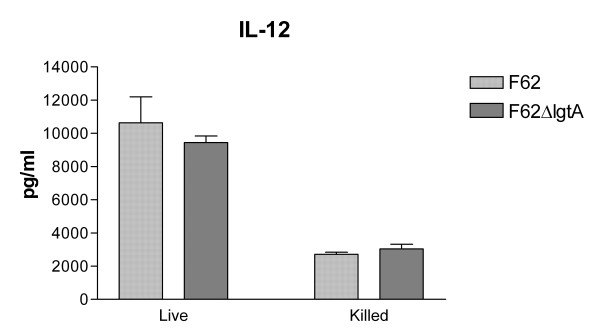
**Production of IL-12 upon challenge with live vs. gentamicin-killed gonococci**. THP-1 cells (1 × 10^6^/ml) were challenged with either 1 × 10^5^/ml live or gentamicin-killed gonococci. At 18 hrs post challenge, IL-12 was measured by ELISA. These data represent one experiment, performed in triplicate. Error bars indicate the standard deviation.

We hypothesized that the LOS on whole bacteria may be presented differently to monocytes than purified LOS. In order to test this, we challenged THP-1 cells with two variants of live gonococci. IL-12 was measured at 18 hours after challenge with a range of bacterial doses and levels were shown to be equal between variants (Fig. 3). Similarly, no difference in TNFα was observed between challenge phenotypes (Fig. 4). Challenge with whole-cell bacteria however, did result in greater production of TNFα when compared with the molar equivalent challenge dose of LOS. The same trend was evident for IL-12 (data not shown). Since this observation was likely to have resulted from growth of the bacteria *in vitro*, we eliminated this variable by using gentamicin sulfate to kill the gonococci prior to challenge. Gentamicin is an aminoglycoside antibiotic and binds directly to ribosomal RNA, thereby inhibiting protein synthesis [32]. Use of this antibiotic does not cause lysis of the bacteria. Upon staining and visualization of killed bacteria, the cells appeared as normal diplococci (data not shown). As shown in figure 5, killed gonococci elicited less IL-12 than live gonococci at 18 hrs post challenge. This is likely due to increased concentrations of bacterial surface components due to bacterial growth. No differences were observed between LOS variants. To avoid the complication of *in vitro *gonococcal growth as well as phase variation, we chose to utilize gentamicin-killed bacteria for the rest of the experiments described.

### Challenge of primary human monocytes with two whole-cell LOS variants reveals similar elicitation of TNFα, IL-10, IL-12, and IL-8

**Figure 6:**
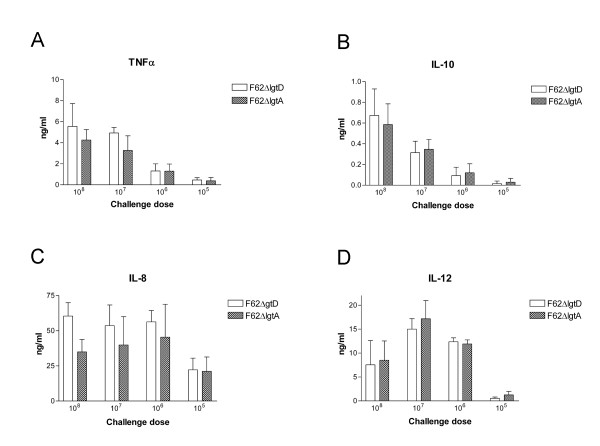
**Production of TNFα, IL-10, IL-12, and IL-8 upon challenge of primary human monocytes**. Monocytes were isolated from human whole blood and cells (1 × 10^6^/ml) were challenged with varying doses of gentamicin-killed gonococci. Sample supernatants were collected at 18 hrs post challenge and cytokines were analyzed by ELISA. Clear bars indicate challenge with F62ΔlgtD and shaded bars indicate challenge with F62ΔlgtA Challenge with media alone resulted in no measurable TNFα or IL-10 and less than 100 pg/ml IL-12; Approximately 1 ng/ml IL-8 was measurable from negative controls, with a standard error of 0.8 ng/ml. (A) TNFα (B) IL-10 (C) IL-8 (D) IL-12 These data represent three experiments, each performed in triplicate. Error bars indicate the standard error of the mean.

In order to examine the effects of LOS variation in a primary cell model, we utilized monocytes derived from human whole blood. These primary cells were challenged with two variants of gentamicin-killed gonococci, F62ΔlgtA (as described above) and F62ΔlgtD which expresses a single LOS component containing the terminal lacto-*N*-neotetraose structure [9]. Use of these fixed variants also ensured that no variation of LOS was occurring *in vitro*. After incubation, sample supernatants were tested for the presence of TNFα, IL-10, IL-12, and IL-8. Over the dose range tested, no differences in cytokine production were observed when comparing the two variants (Fig. 6). However, these data demonstrate that gonococci can elicit all four of these cytokines from human monocytes. In addition, a large amount of the chemokine IL-8 was elicited by gonococci, implying a significant role for this chemokine during infection by *N. gonorrhoeae*.

### Sialylation of gonococcal LOS does not alter production of TNFα or IL-12

**Figure 7:**
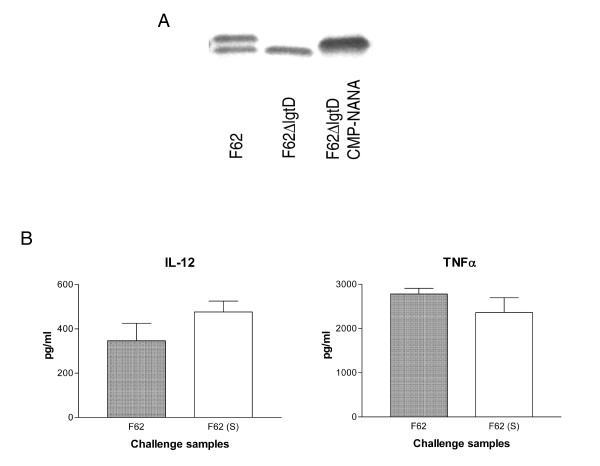
**Production of TNFα and IL-12 upon challenge with gonococci expressing sialylated LOS**. Bacterial LOS was analyzed via polyacrylamide gel electrophoresis. (A) LOS samples shown (from left): wild type F62, F62ΔlgtD, and F62ΔlgtD grown in the presence of CMP-NANA (sialylated). (B) THP-1 cells (1 × 10^6^/ml) were challenged with sialylated and unsialylated *N. gonorrhoeae*. Cytokine production in sample supernatants was measured by ELISA. Left panel: IL-12 levels after an 18 hour challenge of THP-1 cells (1 × 10^6^/ml) with 1 × 10^2^/ml live *N. gonorrhoeae*. Right panel: TNFα levels after 7 hour challenge of THP-1 cells (1 × 10^6^/ml) with 1 × 10^8 ^gentamicin-killed gonococci. (S) Represents sialylated samples (grown in the presence of CMP-NANA). Challenge with media alone did not result in measurable production of IL-12 or TNFα.

Since sialylation of gonococcal LOS is likely to play a role in natural infection, we examined its effects on monocytic cytokine production. After growing gonococci in the presence of CMP-NANA, the bacterial LOS was extracted and analyzed via polyacrylamide gel electrophoresis. This process confirmed the efficacy of the sialylation procedure. Figure 7A shows (from left) wild type F62 and the F62ΔlgtD gonococcal variant expressing only the fixed lacto-*N*-neotetraose LOS structure, and sialylated F62ΔlgtD (grown in the presence of CMP-NANA). A shift in molecular weight between the F62ΔlgtD and sialylated F62ΔlgtD samples is indicative of the addition of sialic acid to the terminal LOS sugar. Figure 7B shows the result of two challenge experiments utilizing the sialylated gonococci. There was no significant difference observed in either IL-12 or TNFα production as a result of challenge with sialylated bacteria. Therefore sialylation of gonococcal LOS does not appear to play a role in altering host production of TNFα or IL-12.

### Gonococcal sialylation elicits production of MCP-2

**Figure 8:**
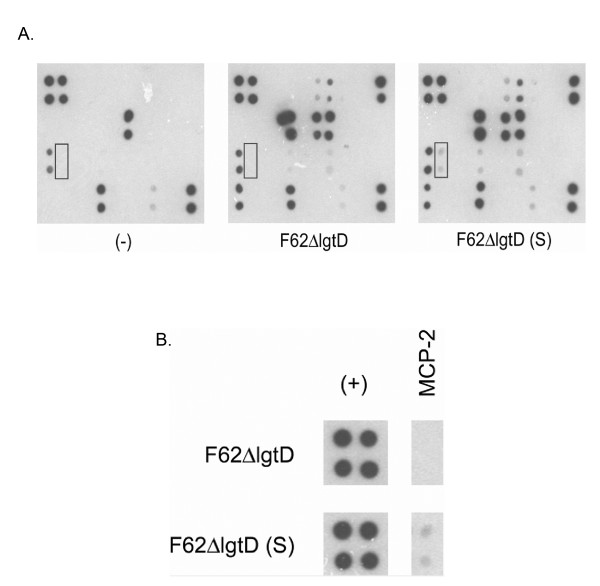
**Comparison of MCP-2 production upon challenge with either F62ΔlgtD or sialylated F62ΔlgtD**. Primary human monocytes (1 × 10^6^/ml) were challenged with gentamicin-killed gonococci (1 × 10^7^/ml) for 18 hours prior to analysis of sample supernatants using a protein array to detect the presence of cytokines. Densitometric analysis was carried out using a Gel Doc 2000 camera and Quantity One Software (BioRad Laboratories, Inc.). Analysis of internal positive controls (+) among array exposures using a paired, two-tailed, t test with a 95% confidence interval indicated that the means were statistically similar (P > 0.05), allowing cross comparison of sample cytokine levels. (A) Whole array for each sample: (-) negative control, F62ΔlgtD, and F62ΔlgtD (S) Dashed boxes indicate the location of MCP-2 antibodies spotted on the array membrane. (B) Internal positive controls compared with MCP-2. Data shown are from one experiment. (S) Indicates sialylated samples.

In order to further study whether sialylation of gonococcal LOS alters monocytic cytokine production, we utilized a cytokine array (Fig. 8A). This assay allowed simultaneous measurement of 42 different cytokines. Challenge with gonococci resulted in upregulation of several cytokines including: GRO (growth regulated oncogene), IL-1β, IL-6, IL-10, GM-CSF (granulocyte-macrophage colony stimulating factor), TNFα, MDC (macrophage-derived chemoattractant), and RANTES when compared to samples challenged with media alone. Several cytokines [IL-8, MCP-1, and PDGF-B (platelet-derived growth factor-B)] were also measurable in unchallenged samples. There is a slight disconnect between cytokines measured in this assay compared with those measured previously by ELISA. No IL-12 was measurable during the array experiment and we attribute this to the different antibody pairs used in printing the array. In addition, IL-8 did not appear to be significantly upregulated between unchallenged and challenged samples since the spots were saturated on both arrays chosen for analysis. Even upon examination of a one second exposure, values for the IL-8 spots far exceeded positive control values. In addition, positive control values from these early exposures did not fall in the linear range, and therefore these arrays could not be included in the statistical analysis (data not shown). However, previous analysis by ELISA did allow quantitation of the IL-8 produced during challenge.

Interestingly, challenge with sialylated gonococci caused production of MCP-2 (monocyte chemoattractant protein-2) in comparison with unsialylated gonococci (Fig. 8). MCP-2 was not detected in unchallenged samples or those challenged with unsialylated gonococci. Due to the limits of this type of assay, we could not determine fold change in the observed difference in MCP-2 production. A variety of immune cells are targets for this chemokine and chemotactic responses have been shown to differ depending upon the concentration of MCP-2 *in vitro*. Quantitation of MCP-2 regarding these gonococcal challenge experiments would allow greater speculation as to its biological significance [33].

## Discussion

Gonococcal LOS is an important surface molecule that contributes to disease. In this study, we measured the effect of variation in carbohydrate structure on the production of a variety of cytokines by monocytes. The data demonstrate that alterations in the carbohydrate moiety of LOS do not impact the production of most cytokines by human monocytes. However, whole-cell bacterial challenge was more stimulatory than challenge with purified LOS, implying an important role for other surface structures with regard to cytokine elicitation. Sialylation of LOS resulted in elicitation of the chemokine MCP-2 from challenged cells. As the LOS structure associated with symptomatic infection (lacto-*N*-neotetraose) is readily sialylated *in vivo*, differential elicitation of cytokines due to sialylation could significantly impact disease progression by increasing the response of specific immune cell types.

Several groups have measured cytokine production during *in vitro *challenge with *N. gonorrhoeae*. Because different models have been employed, the results vary with respect to which cytokines are produced and in what quantities. In this study, we observed production of TNFα, IL-12, IL-10, and IL-8 by monocytes in response to whole-cell gonococcal challenge as well as in response to challenge with purified LOS. Intact gonococci elicited a much greater response than purified LOS/LPS. Since these experiments were performed, this observation has been confirmed by several groups studying *N. meningitidis*. It has been demonstrated that *N. meningitidis *deficient in detectable LOS (LpxA^-^) induces substantial cytokine production from human peripheral blood mononuclear cells when compared to wild type bacteria [34,35]. Similarly, intact meningococci have been shown to be more potent inducers of several cytokines than equal amounts of purified LPS [34]. In contrast, Unkmeir, *et al*. reported that challenge of dendritic cells with an *N. meningitidis *strain lacking LOS resulted in almost undetectable levels of TNFα, IL-6, and IL-8 [36]. However, this difference may be related to the use of dendritic cells as a model. These data, along with our observations, indicate that a substantial portion of the cytokines elicited from gonococcal interactions with monocytes, result from non-LPS components.

During these experiments we observed no difference in the elicitation of TNFα, IL-10, IL-12, or IL-8 between gonococcal LOS variants. Based on these observations, we believe that while the carbohydrate portion of the LOS molecule is important for pathogenesis of the organism, it does not play a significant role in determining host cytokine profile during gonococcal encounters with monocytes. Pridmore, *et al*. challenged THP-1 cells with purified LOS (200 ng) from different neisserial strains and measured TNFα production and signaling through TLR4/MD2 [35]. Some variability was observed, although this did not correspond with oligosaccharide structure of the strains. Braun, *et al*. demonstrated that *N. meningitidis *variants expressing LOS immunotypes isolated from diseased patients induced significantly higher levels of TNFα and IL-6 when compared with immunotypes primarily associated with carriers of the bacteria [37]. However, the immunotypes that differed in their ability to induce cytokine production each expressed identical oligosaccharide moieties. Interestingly, disruption of the lpxLII gene in *N. gonorrhoeae *was shown to alter the lipid A backbone of the LOS molecule, resulting in mutants with a reduced ability to induce TNFα, IL-1β, IL-6, and IL-8 from a human macrophage model [38].

Taken together, these data imply that differences in the immunostimulatory potential of gonococcal LOS variants during interactions with human monocytes originate from alterations in the lipid A structure. This hypothesis is supported by the fact that acylation patterns of the lipid A portion of meningococcal LOS have been demonstrated to be important in determining levels of TNFα elicited from a human macrophage cell line [39].

It has been previously shown that when dendritic cells are challenged with *N. meningitidis*, optimal production of TNFα and Il-12 only occurs upon internalization of the bacteria [27]. In addition, this internalization appeared to be dependent on expression of LOS, as an LOS-deficient mutant was internalized poorly compared to wild type bacteria. In future studies, it would be interesting to study the effects of gonococcal oligosaccharide alterations during interactions with other cellular models, such as dendritic cells. In addition, measurement of internalization, as well as cytokine production during challenge of human macrophages with gonococcal lipid A mutants, may provide significant insight into gonococcal pathogenesis.

Data collected from these experiments allowed a broad look at the cytokine profile of monocytes after gonococcal challenge. A variety of cytokines and chemokines were measurable during infection including: GRO, IL-1β, IL-6, IL-10, IL-12, GM-CSF, TNFα, MDC, and IL-8. The cytokine profile we observed differed slightly from that observed by Makepeace, *et al*., who utilized a differentiated human macrophage model during gonococcal challenge studies [40] Though production of several cytokines [including TNFα, IL-6, macrophage inflammatory protein-1-alpha (MIP-1α), and RANTES] was measured, no secretion of IL-1β, epithelial neutrophil-activating protein 78 (ENA-78), GM-CSF, IL-10, or IL-12 was reported. Since we were able to measure IL-1β, GM-CSF, IL-10 and IL-12 during challenge, it is possible that use of differentiated cells, a different gonococcal strain (P9), and a higher infectious dose (MOI = 400) by this group explains these differences with regard to our own observations. Further analysis of the roles of the cytokines and chemokines measured in this study could significantly contribute to our understanding of gonococcal disease.

Sialylation of gonococcal LOS has been previously shown to convert serum sensitive strains to a serum resistant phenotype [41,42]. However, sialylation has also been shown to play a role in other aspects of gonococcal pathogenesis including interactions with antibodies and phagocytes (for review see [43]). In these studies, sialylation of gonococcal LOS prior to challenge of THP-1 cells resulted in similar production of TNFα or IL-12. These experiments were followed by use of an array to examine cytokine production from challenged primary monocytes. This work demonstrated that sialylated gonococcal challenge samples exhibited upregulation of monocyte chemoattractant protein-2 (MCP-2), in comparison to unchallenged samples and those challenged with unsialylated bacteria. MCP-2 is a low molecular weight monocyte chemotactic cytokine which is closely related to MCP-1 and monocyte chemoattractant protein-3 (MCP-3) [33]. Target cells for MCP-2 include monocytes, T lymphocytes, natural killer cells, eosinophils, and basophils, though monocytes are the most sensitive. The concentration of MCP-2 present *in vitro *has been shown to differentially activate specific target cell populations[33]. While MCP-2 production in response to gonococcal infection was seen in a human mononuclear cell model, gonococci were not sialylated prior to challenge [44]. As LOS molecules associated with symptomatic infection and increased invasiveness are readily sialylated *in vivo*, production of MCP-2 during infection may substantially increase the response of a specific cellular population during gonococcal infection. Future determination as to the quantities of MCP-2 produced during monocyte interactions with sialylated gonococci may provide insight into the pathogenesis of these more virulent strains. In addition, further analysis of the mechanism by which sialylated LOS causes production of MCP-2 may significantly add to our knowledge of the pathogenesis of *N. gonorrhoeae*.

## Conclusion

In order to analyze the impact of antigenic variation in gonococcal LOS, we utilized gonococcal mutants with specific LOS structures to challenge human monocytes. These mutants expressed LOS structures associated with either symptomatic (F62/F62ΔlgtD) or asymptomatic (F62ΔlgtA) infection. These specific LOS structures have been previously shown to affect gonococcal interaction with epithelial cell models [9]. Upon challenge of monocytes during this study, the levels of several proinflammatory cytokines were measured. The data generated demonstrate that alterations in the carbohydrate moiety of LOS do not directly impact the production of these cytokines. From our data, as well as several other reports, it seems likely that differences in the immunostimulatory potential of LOS variants during interactions with human monocytes may stem from alterations in the structure of lipid A [35,37-39]. In addition, whole-cell bacterial challenge was shown to be much more stimulatory than challenge with purified LOS. This implies that other surface structures may play an important role with regard to cytokine elicitation. Sialylation of LOS was shown to result in upregulation of the chemokine MCP-2 from challenged cells. As MCP-2 is associated with recruitment of monocytes, this finding may indicate a role for sialylation during further interactions with this cell type. A greater understanding of the mechanism behind MCP-2 elicitation by gonococci, as well as quantitation of this cytokine during challenge, will be important questions to explore in the future.

## Methods

### Bacterial strains and infection

Gonococcal strain F62 was obtained from Dr. P. Frederick Sparling (University of North Carolina, Chapel Hill, NC). F62ΔlgtD, a derivative of F62 that expresses a non-varying LOS, lacto-*N*-neotetraose, and F62ΔlgtA, a derivative of F62, expresses a fixed lactosyl LOS structure and has been previously described [9]. Prior to each experimental challenge, gonococci were grown on GCK agar and piliated, Opa^- ^organisms were selected. (Initial studies indicated that non-piliated gonococci elicited a similar cytokine profile compared with piliated gonococci, regarding measurement of TNFα, IL-8, IL-12, and IL-10.) Bacteria were suspended in GCP broth supplemented with Kellogg's solution [45], the concentration of cells was determined spectrophotometrically and verified via viable plate count. Cells were killed by incubation for three hours with 2 mg/ml gentamicin sulfate. Bacteria were stored at 4°C until challenge of human cells. Prior to bacterial challenge, gentamicin-killed bacteria were collected by centrifugation (12,000 rpm for 5 min.), resuspended in RPMI 1640 (Mediatech, Herndon, VA), and diluted as needed. Use of killed bacteria prevented growth and phase variation during the incubation period. For experiments involving *E. coli*, either strain DH5-α was used (New England Biolabs, Ipswich, MA) or an Rc strain of *E. coli *(*E. coli *Genetic Stock Center) as indicated. For experiments using LPS as a negative control, LPS was obtained from Sigma Chemical Co. (St. Louis, MO).

### LOS extraction procedure for monocyte challenges

Purified LOS was obtained from broth grown cells through the hot-phenol method followed by lyophilization [46]. Repeated phenol water extractions/lyophilizations were performed until an aliquot of purified LOS dissolved in water at a concentration of 1 mg/ml gave no detectable absorbance, when measured spectrophotometrically at A280. Molar equivalencies were determined by weighing purified LOS, and analyzing dilutions of these samples along with dilutions of LOS prepared using the quick prep procedure via SDS-PAGE. Gels were silver stained, and the intensity of bands obtained with purified LOS were compared to the intensity of bands obtained from quick preps. This allowed us to determine that approximately 10 ng LOS was equivalent to 1 × 10^5 ^gonococci.

### LOS extraction procedure for SDS-PAGE analysis

After sialylation, quick preparations of gonococcal LOS were prepared from bacterial suspensions using the method of Hitchcock and Brown [47]. An aliquot of 1 ml (approximately 1 × 10^9 ^bacteria/ml) was centrifuged at 10,000 rpm and the pellet was re-suspended in 50 μl lysing solution [1 M Tris-HCl (pH = 6.8), 2% SDS, 4% 2-mercaptoethanol, 10% glycerol, bromophenol blue to saturation]. Proteinase K (10 μl of 1 mg/ml stock) was added and the sample was incubated at 60°C for one hour. Samples were then diluted 1:25 in lysing solution. Prior to SDS-PAGE analysis, samples were boiled for 10 min and 5 μl was loaded onto the gel.

### Gonococcal LOS sialylation

Prior to challenge using sialylated bacteria, gonococci were grown on GCK agar and piliated, Opa^- ^organisms were selected. Bacteria were suspended in GCP broth supplemented with Kellogg's solution [45]. Cells were collected from plates using sterile swabs and resuspended in PBS to a Klett reading of 40. The concentration of cells was determined spectrophotometrically and verified via viable plate count. CMP-NANA was added to the bacterial suspension at a concentration of 50 μg/ml. Bacteria were incubated for 4 hrs, in a 37°C rolling incubator. After incubation, the concentration of bacteria was verified by viable plate count. Bacterial sialylation was verified by LOS extraction and SDS-PAGE analysis (as described above).

### Cell lines

THP-1 (human, peripheral blood, leukemia, acute monocytic) cells used were originally from the American Type Culture Collection (Manassas, VA) and were donated to our laboratory courtesy of Dr. David Mosser. Cells were grown in RPMI 1640 with 10% fetal bovine serum (Sigma-Aldrich Chemical Co, St. Louis, Mo) and 2 mM L-glutamine, supplemented with 0.05 mM 2-mercaptoethanol. FBS contained less than 10 EU/ml endotoxins, as measured by USP gel clot assay done by the commercial provider. Fresh medium was added every 2–3 days or when cell concentrations exceeded 8 × 10^5 ^cells/ml. Prior to experimentation, cell numbers were enumerated using a hemocytometer and plated at a concentration of 1 × 10^6 ^per ml.

### Isolation and culture of human monocytes/macrophages

Whole blood was drawn from healthy volunteers and peripheral blood mononuclear cells were prepared by density gradient centrifugation using Ficoll-Paque Plus. Briefly, blood was collected in heparinized tubes (BD Biosciences, San Jose, CA) and transferred to 50 ml conical tubes. The volume in each tube was brought to approximately 36 ml using warm PBS and cells were gently pipeted to ensure mixing. 14 ml of Ficoll-Paque Plus (Amersham Biosciences, Piscataway, NJ) was added beneath the blood suspension. (When necessary an untreated tube was used to collect blood for serum.) All blood tubes were centrifuged for 20 min at room temperature (RT), at 2500 rpm, and with the brake set to zero. This step was followed by transfer of the opaque layer (containing peripheral blood mononuclear cells) to a new 50 ml conical tube and dilution of the cells 1:1 with RPMI 1640 or removal of the serum layer into a separate tube. (Serum was heat inactivated for 25 min at 56°C prior to use in sample media.) Cells were centrifuged for 10 min at 4°C at 1500 rpm. The pellet was resuspended in a small volume of RPMI 1640, transferred to a 15 ml conical tube, and centrifuged for 10 minutes at 4°C, at 2500 rpm. Supernatants were aspirated and cells counted on a hemocytometer. Prior to bacterial challenges, cells were seeded at 1 × 10^6 ^cells/ml in tissue culture-treated plates in RPMI 1640 and incubated at 37°C, 5% CO_2 _with humidity. After 30 min, the cells were washed to remove the nonadherent population and RPMI 1640 (supplemented with 10% autologous heat-inactivated serum) was added preceding bacterial challenge.

All studies employing human blood were approved by the University of Maryland's Institutional Review Board (IRB #01321, "Immune Response to Gonococcal Infection") prior to the initiation of any of these experiments.

### ELISA

Prior to bacterial challenge, primary human monocytes were isolated and seeded as described above. Following challenge with the appropriate dilution (100 μl) of gentamicin-killed gonococci, cells were incubated in RPMI 1640 supplemented with 10 % autologous human serum at 37°C 5 % CO_2 _with humidity for the time specified. Cell supernatants were collected and assayed for the presence of cytokines by sandwich ELISA. Upon collection of supernatants, a protease inhibitor cocktail was added and each sample was stored at -80°C until analysis. Antibody pairs and recombinant standards for human TNFα, IL-8, IL-10, and IL-12p40 were purchased from BD Pharmingen (San Diego, CA). Antibody pairs, recombinant standards, and neutralizing antibody for human IL-1β were purchased from R&D Systems (Minneapolis, MN). ELISAs were carried out according to protocols provided by BD Pharmingen. Streptavidin alkaline phosphatase and p-nitrophenyl phosphate substrate were purchased from Southern Biotech (Birmingham, AL) and used according to the manufacturer's specifications. Samples were read at 405 nm in 96-well, untreated, flat-bottom plates.

### Cytokine array

Primary human monocytes were isolated as described above and seeded into 24-well tissue culture plates at 1 × 10^6 ^cells/ml. The appropriate dilution (100 μl) of gentamicin-killed gonococci was added to each sample monolayer, and cells were incubated in RPMI 1640 supplemented with 10 % autologous human serum at 37°C, 5 % CO_2 _with humidity for 18 hrs. Supernatants from quadruplicate samples were pooled before addition to the array membrane. The RayBio Human Cytokine Array III was purchased from Raybiotech, Inc. (Norcross, GA) and used according to manufacturer's instructions. Array membranes were analyzed using a Gel Doc camera and Quantity One software (Bio-Rad Laboratories, Inc., Hercules, CA). The volume array tool (for 96 wells) was used to specify area intensity for each spot within an image. A "background" volume was placed over the internal negative control for each array. The average intensity of pixels in this volume was calculated and subtracted from each pixel in all other volumes. This process resulted in calculation of the "adjusted volume" (i.e. spot intensity minus the average intensity of the internal negative control). Positive controls from several exposures were compared and the means were found to be statistically similar (P > 0.05) using a paired, two-tailed, t test with a 95% confidence interval, thus allowing cross comparison of sample cytokine levels. Adjusted volumes (two for each cytokine) were then averaged and the standard deviation was determined.

## List of Abbreviations Used

ASGP asialoglycoprotein

CMP-NANA cytidine 5'-monophospho-*N*-acetylneuraminic acid

ENA-78 epithelial neutrophil-activating protein 78

GM-CSF granulocyte-macrophage colony stimulating factor

GRO growth regulated oncogene

IL-1β interleukin-1 beta

IL-4 interleukin-4

IL-6 interleukin-6

IL-8 interleukin-8

IL-10 interleukin-10

IL-12 interleukin-12

LOS lipooligosaccharide

LPS lipopolysaccharide

MCP-1 monocyte chemoattractant protein-1

MCP-2 monocyte chemoattractant protein-2

MCP-3 monocyte chemoattractant protein-3

MDC macrophage-derived chemoattractant

MIP-1α macrophage inflammatory protein-1-alpha

Opa opacity-associated protein

PDGF-B platelet-derived growth factor-B

PID pelvic inflammatory disease

RANTES regulated upon activation, normal T-cell expressed, and presumably secreted

TNFα tumor necrosis factor alpha

## Authors' contributions

JP performed all of the experimental manipulations described in this manuscript. DS conceived of the study, and participated in its design and coordination. All authors read and approved the final manuscript.
